# Patient Satisfaction with HIV/AIDS Care and Treatment in the Decentralization of Services Delivery in Vietnam

**DOI:** 10.1371/journal.pone.0046680

**Published:** 2012-10-05

**Authors:** Bach Xuan Tran, Nhung Phuong Thi Nguyen

**Affiliations:** 1 Institute for Preventive Medicine and Public Health, Hanoi Medical University, Hanoi, Vietnam; 2 School of Public Health, University of Alberta, Edmonton, Alberta, Canada; 3 School of Public Health, University of Texas at Houston, Houston, Texas, United States of America; University of Ottawa, Canada

## Abstract

**Objective:**

We evaluated the patient satisfaction with HIV/AIDS care and treatment and its determinants across levels of health service administration in Vietnam.

**Methods:**

We interviewed 1016 patients at 7 hospitals and health centers in three epicenters, including Hanoi, Hai Phong, and Ho Chi Minh City. The Satisfaction with HIV/AIDS Treatment Interview Scale (SATIS) was developed, and 3 dimensions were constructed using factor analysis, namely “Quality and Convenience”; “Availability and Responsiveness”; and “Competence of health care workers”.

**Results:**

In a band score of (0; 10), the mean scores of all domains were large; it was the highest in “Competence of health workers” (9.34±0.84), and the lowest in “Quality and Convenience” (9.03±1.04). The percentages of respondents completely satisfied with overall service quality and treatment outcomes were 42.4% and 18.8%, respectively. In multivariate analysis, factors related to higher satisfaction included female sex, older age, and living with spouses or partners. Meanwhile, lower satisfaction was found among patients who were attending provincial and district clinics; in the richest group; had higher CD4 count; and drug users.

**Conclusion:**

This study highlights the importance of improving the quality of HIV/AIDS services at the provincial and district clinics. Potential strategies include capacity building for health workers, integrative service delivery, engagements of family members in treatment supports, and additional attention and comprehensive care for drug users with HIV/AIDS.

## Introduction

Over the past decade, the rapid expansion of antiretroviral treatment (ART) in Africa and Asia has dramatically reduced HIV-related morbidity and mortality, and transformed HIV into a chronic illness [1]. However, it remains a challenge to achieve the universal access target and ensure the quality of HIV/AIDS care and treatment services in many low-income countries with the hardest hit of HIV epidemics [2]–[4]. Delayed access, suboptimal adherence, low retention rate, and poor outcomes of ART have been observed in these settings, especially in large drug injection-driven HIV epidemics [2], [5], [6]. Recently, the UNAIDS Secretariat and WHO launched Treatment 2.0, an initiative designed to achieve and sustain universal access and maximize the preventive benefits of ART [1]. Treatment 2.0 promotes the decentralization and integration of ART with other HIV and non-HIV health services in order to bring ART closer to patients, reduce financial, distance, and administrative barriers, and engage community in delivering treatment supports [1], [7]. To successfully adopt this model, understanding of contextual influences to services' quality and utilization across levels of health service administration is of great interest to program managers and policy makers, especially in resource-limited settings [1], [7].

Since 2011, Vietnam has been taking the lead in universal access to comprehensive HIV services as the first country pilots the Treatment 2.0 [7]. The HIV epidemic in Vietnam is still in a concentrated stage which has been largely driven by drug injection. By 2010, there were 197,335 HIV-positive cases managed in the country, of these, 70% had a history of drug injection. The HIV prevalence was very high among most-at-risk populations, for instance, injecting drug users (13.4%), female sex workers (3%), and men who have sex with men (16.7%) [8]. Over the past five year, the coverage of ART for HIV/AIDS patients in need of treatment has increased exponentially to 50% by 2010 (59,000 HIV/AIDS patients were treated), approximately 18 times the ART coverage in 2005 [8]. A total of 318 ART clinics has been established in the country, of those 52% at the district level, 43% at the provincial level and 5% at the central level [8]. Rapid scale-up and decentralization of ART services promise substantial benefits to HIV/AIDS patients; however, at the same time, concerns are raised about the quality and outcomes of these services. Up to now, there is very little experience with ART service delivery as well as patients' preference and reflections in the Vietnamese setting.

In developed countries, measuring patient-reported outcomes and satisfaction is central to designing and evaluating modern healthcare services and delivery system [9]. Patient satisfaction with health care reflects the quality of services from the patients' perspective that supplements to traditional indicators such as survival outcomes or processes of care [9]. In addition, measurements of patient satisfaction could help evaluate the performance of health service delivery, identify patients who need additional attentions or targeted interventions, and predict treatment adherence and outcomes [10]. In developing countries, studies on patients' satisfaction with HIV/AIDS treatment have been growing [11]–[13]. Levels of satisfaction and associated factors varied across measures, sub-groups of patients, clinical stages, clinics, regions, and health care systems, making it essential to characterize these attributes in each setting [14]–[20]. In the present study, a specific instrument for measuring patient satisfaction was developed. Using this measure, we aimed to assess the patient satisfaction with HIV/AIDS care and treatment services, and determined the associations between socioeconomic and clinical characteristics and satisfaction among HIV/AIDS patients in Vietnam.

## Methods

### Ethics Statement

Data use was approved by the Authority of HIV/AIDS Control, Ministry of Health of Vietnam. Ethical approval was granted by the University of Alberta's Health Research Ethics Board. Written informed consent was obtained from all participants after clearly introducing the survey. Respondents could refuse to participate or withdraw from the interview at any time, and this did not affect their continuation of health care services. Confidentiality was assured using codes of patient's information, and secured storage was prepared for both paper questionnaires and electronic dataset.

### Study settings

The 2012 Vietnam HIV Services Users Survey (HSUS) was conducted in three epicenters of Vietnam, including Ha Noi, Hai Phong, and Ho Chi Minh City [21]. These metropolitan areas represent different geographical regions with the largest HIV epidemics in Vietnam. Ha Noi is the capital of Vietnam with a population of 6 millions, among those, approximately 18,000 people living with HIV. Of 28 ART clinics in Hanoi, there were 2 central, 11 provincial and 15 district sites. Hai Phong, is a port city in northern Vietnam of about 1.8 million citizens. There were 6930 HIV-positive people, 3 provincial and 10 district ART clinics. The biggest southern metropolitan center is Ho Chi Minh City. It has the largest HIV-positive population nationwide with 46,507 cases over 8 million residents. It has 32 ART clinics, including 21 provincial and 11 district sites [22].

### Study design and participant recruitment

We conducted a cross-sectional survey in 1016 patients at 07 ART clinics (17% of the total HIV cases), including 1 central hospital with 201 patients (20%), 3 provincial hospitals with 406 patients (40%), and 3 district health centers with 409 patients (40%). Eligible subjects included those patients who were diagnosed HIV-positive, registered for HIV/AIDS care or treatment at selected sites, and were present at the clinics during the period of the study. This included both inpatients and outpatients who came for receiving regular check-ups and ARV medications. Selection of patients was basically convenient given the fact that we could not develop a sample frame of HIV/AIDS individuals due to confidentiality. There was only the total number of patients managed at each hospital and province. The proportion of sample by cities was 60% from Hanoi, 20% from Hai Phong, and 20% from Ho Chi Minh City. Well-trained investigators came to each HIV/AIDS out-patient or in-patient department, clearly explained the purposes and study information, and invited patients to participate in the study. To reduce information bias, these interviewers were not from the same hospital where the patients were approached.

### Measures and Instrument

In face-to-face interviews, we collected information about socioeconomic and HIV-related characteristics of patients and their satisfaction with HIV/AIDS treatment services. The socioeconomic characteristics included age, gender, marital status, religion, education level, employment, and income. Household's monthly income was self-reported by summing up all sources of income from each member in 2011 and then stratified into five quintiles. As for clinical characteristic, HIV/AIDS stages, history of drug use, the duration of knowing their HIV status, and duration of ART were recorded.

**Development of the Satisfaction with HIV/AIDS Treatment Interview Scale (SATIS):** We developed a measure to assess patients' satisfaction, namely the Satisfaction with HIV/AIDS Treatment Interview Scale (SATIS) following a standard procedure. First, a systematic review of the English literature identified from PubMed searches was conducted to explore candidate measure items of patient satisfaction with HIV/AIDS care and treatment services [11], [17], [20], [23]–[28]. We found a number of instruments, which were applied in both developed and developing countries. Basically, the breadth of measurements included the following dimensions: 1) Technical quality and competence, 2) Facilities and equipment, 3) Interpersonal skills, 4) Communication and guidance, 5) Financial aspects, 6) Waiting and spending time, 7) Services availability, accessibility, and convenience, and 8) Confidentiality. Secondly, we did three focus group discussions with HIV/AIDS patients, health workers and managers, and researchers to incorporate their diverse perspectives and improve the face validity of the measure. Lastly, we constructed a pooled list of measure items and then shortened it based on items' importance and relevance justified by researchers and health workers. We piloted the instrument in a small convenient sample to examine if any cultural, language, and administration methods should be revisited. Finally, the SATIS included 10 questions to be surveyed. The response options included a band score of (0–10) where 0 and 10 indicate a complete unsatisfaction and a complete satisfaction, respectively. Domains scores were estimated by averaging the score of all domain items. The higher SATIS scores indicated higher levels of patient satisfaction with HIV/AIDS care and treatment services. Besides, we employed two global rating questions of general satisfaction with health services and treatment outcomes.

### Statistical analysis

*Exploratory factor analysis* was applied to examine the construct validity of the SATIS measurement. These factors were extracted by the principle component analysis at an eigenvalue of 1.0, which was the threshold, defined using the scree test, where the eigenvalue curve flattened out. Orthogonal Varimax rotation with Kaisers' normalization was used to reclassify the measure items in order to increase the interpretability of these factors. The cut-off point for factor loadings was set at 0.40. We had a cross-loading in one item and assigned it to the corresponding domain based on the nature of the questions and the overarching dimension. Internal consistency reliability of measurement was estimated using Cronbach's alpha. Chi-square and ANOVA tests were used to examine the differences between proportions or means. *Multivariable linear regression* was used to determine factors associated with general satisfaction. We applied a stepwise forward model building strategy which selected variables based on the log-likelihood ratio test at a p-value<0.1, and excluded variables at a p-value>0.2 [29]. The level of signification was set at a p-value of less than 0.05.

## Results

### Socio-demographic and clinical characteristics of respondents

The baseline demographic characteristics of respondents were summarized in Table 1. Participants in the study were at an average age of 35.4 years (sd = 7.0), and the majority of them were male (63.8%). The percentage of subjects with high-school level of education attainment was 63.0% at the central clinic, which was higher than that of the provincial (43.6%) and district levels (35.2%). Most of the respondents got married or lived with their partners (64%). More than 70% of patients were unemployed or had unstable jobs. Notably, a higher proportion of the richest group (28.4%) was seen at the central clinic relative to that of other health system levels. About half of the sample were drug users (46.1%), among them, 87.2% were still using drug and 11.9% taking Methadone Maintenance Treatment (MMT). Patients were diagnosed HIV-positive for an average length of 5 years; 37% were at AIDS stage. The mean duration of taking ART was 3 years; slightly longer at the central level than the provincial and district levels. Percentage of patients who had a CD4 count less than 200 cells/ml was as high as 32.9% at the central level, 36.3% at the provincial level and 25.1% at the district level.

**Table 1:** Patient characteristics by levels of health system administration.

	Total		Levels of the health system						p-value
			Central		Provincial		District		
	Mean	SD	Mean	SD	Mean	SD	Mean	SD	
**Age**	35.4	7.0	36.0	6.8	35.1	7.3	35.3	6.7	0.15
**Years living with HIV**	5.4	3.5	5.8	3.6	5.4	3.6	5.3	3.3	0.26
**Years taking ART**	3.0	2.1	3.5	2.2	2.9	2.1	3.0	2.0	0.44
**Sex** (Male)	648	63.8	122	60.7	258	63.6	268	65.5	0.50
	N	%	N	%	N	%	N	%	
**Education**									
Below high school	556	54.7	62	30.9	229	56.4	265	64.8	<0.01
High school and above	460	45.3	139	69.2	177	43.6	144	35.2	
**Marital status**									
Single	142	14.0	28	13.9	72	17.7	42	10.3	<0.01
Live with spouse/part	650	64.0	147	73.1	248	61.1	255	62.4	
Widow(er), Divorced, Separated	224	22.1	26	12.9	86	21.2	112	27.4	
**Employment**									
Unemployed	181	17.8	32	15.9	68	16.8	81	19.8	<0.01
Free lancer	534	52.6	73	36.3	221	54.4	240	58.7	
Stable jobs	207	20.4	80	39.8	78	19.2	49	12.0	
Others	94	9.3	16	8.0	39	9.6	39	9.5	
**Religion**									
None	761	74.9	179	89.1	288	70.9	294	71.9	<0.01
Buddhism	206	20.3	17	8.5	92	22.7	97	23.7	
Others	49	4.8	5	2.5	26	6.4	18	4.4	
**Income quintiles**									
Poorest	184	20.0	35	19.1	92	24.2	57	16.0	<0.01
Poor	166	18.0	27	14.8	80	21.0	59	16.6	
Middle	192	20.9	37	20.2	77	20.2	78	21.9	
Rich	194	21.1	32	17.5	68	17.9	94	26.4	
Richest	184	20.0	52	28.4	64	16.8	68	19.1	
**HIV/AIDS stages**									
Asymptomatic	126	12.4	32	15.9	44	10.8	50	12.2	<0.01
Symptomatic	508	50.0	126	62.7	179	44.1	203	49.6	
AIDS	382	37.6	43	21.4	183	45.1	156	38.1	
**CD4 cell count**									
< = 200	249	31.1	53	32.9	115	36.3	81	25.1	0.01
200<cd4< = 350	249	31.1	52	32.3	102	32.2	95	29.4	
350<cd4< = 500	194	24.2	40	24.8	66	20.8	88	27.2	
>500	109	13.6	16	9.9	34	10.7	59	18.3	
**Drug use**	468	46.1	85	42.3	170	41.9	213	52.1	0.01
**Taking MMT**	121	11.9	12	6.0	24	5.9	85	20.8	<0.01

### Measurement properties of the SATIS

Table 2 shows the construct validity and reliability of the SATIS measurement. Three dimensions emerged from factor analysis that accounted for 84.4% of the variance, namely “Service Quality and Convenience”, “Availability and Responsiveness”, and “Competence of health care workers”. The first factor accounted for 63.3% of the variance. Cronbach's alpha was good to excellent across domains, ranging at (0.74; 0.94) (Table 2). The convergent validity of SATIS dimensions was good and fair in correlation with the “general satisfaction with health services” and “general satisfaction with treatment outcomes”, respectively.

**Table 2:** Factor loading, convergent validity and reliability of the Vietnamese Satisfaction with HIV/AIDS Treatment Interview Scale (SATIS).

		Factor loadings of SATIS domains		
Items	% completely satisfied	Services quality and convenience	Availability and Responsiveness	Competence of health workers
1. Medical confidentiality and respect of patients' privacy	60.1			0.93
2. Competence of health care workers	52.6			0.41
3. Consultation, explanation, and guidance of health care workers	52.5	0.66		
4. Responsiveness of health care workers to patients' questions and requests	51.4		0.68	
5. Quality of ART services delivery	50.8	0.88		
6. Availability of patients' needed health care services	47.7		0.85	
7. Access to information and guidance on hospital services and procedures	47.1	**0.77**	0.44	
8. Inter-professional and inter-departmental collaborations	45.6		0.49	
9. Convenience in check-up booking, waiting time, and administrative procedure	44.6	0.70		
10. Convenience in using medical services	44.2	0.70		
**% floor**		0.0%	0.0%	0.0%
**% ceiling**		37.1%	41.2%	50.9%
**Convergent validity**				
General satisfaction with health services	42.4	0.69	0.71	0.64
General satisfaction with treatment outcomes	18.8	0.44	0.47	0.40
**Reliability**				
Cronbach's alpha		0.94	0.89	0.74
**SATIS domains scores**				
Mean		9.03	9.05	9.34
SD		1.04	1.04	0.84

### Patient satisfaction with HIV/AIDS care and treatment services and its correlated factors

The proportion of respondents who were completely satisfied with different criteria of service quality was the lowest in “Convenience in using medical services” (44.2%) and “Convenience in check-up booking, waiting time, and administrative procedure” (44.6%). Meanwhile, it was the highest in “Competence of health care workers” (52.6%) and “Confidentiality” (60.1%). The average domain score was large across three domains; it was the highest in “Competence of health care workers” (9.34±0.84), and the lowest in “Quality and Convenience” (9.03±1.04) (Table 2). The percentage of respondents completely satisfied with overall service quality and treatment outcomes was 42.4% and 18.8%, respectively.

Table 3 presents the associations between patient's characteristics and satisfaction with different health-care dimensions. In reduced models, levels of the health system were significantly associated with all SATIS domains (p<0.01). Compared to the central level, the magnitude of differences in SATIS domains scores of the provincial and district level was (0.40→0.56) and (0.61→0.68) times the standard deviation of the SATIS domains scores, corresponding to “medium to large” decrements. Moreover, at lower levels of the health system, we found significantly smaller likelihoods of being completely satisfied with both service quality and treatment outcome (p<0.01). Some individual characteristics of patients were also associated with SATIS domain scores. Older patients had higher satisfaction consistently across 3 aspects of the health care service quality (β = 0.01, p<0.05). Lower satisfaction with “Quality and Convenience” was found in men, the richest group (versus the poorest), and in patients who had higher CD4 cell counts. As for services' availability and responsiveness, we found small decrements in this domain score among patients who were male, single, at advanced HIV stages, taking MMT, and drug users. However, it was only statistically significant in the drug use group.

**Table 3:** Factors associated with Patient satisfaction with HIV/AIDS treatment.

	Coefficients of SATIS domain scores				OR of complete satisfaction with	
	Overall	Quality and Convenience	Availability and Responsiveness	Competence of health workers	Service quality	Treatment outcome
**Health system levels** (aCentral)						
Provincial	−0.49	−0.58	−0.55	−0.34	0.27	0.35
	(−0.66 ; −0.31)**	(−0.78; −0.38)**	(−0.75 ; −0.35)**	(−0.50; −0.19)**	(0.18; 0.42)**	(0.22; 0.55)**
District	−0.63	−0.64	−0.71	−0.51	0.24	0.17
	(−0.80; −0.45)**	(−0.84; −0.43)**	(−0.91; −0.50)**	(−0.66; −0.35)**	(0.16; 0.37)**	(0.10; 0.29)**
**Age** (years)	0.01	0.01	0.01	0.01		
	(0.00; 0.02)*	(0.00; 0.02)*	(0.00; 0.02)*	(0.00; 0.02)*		
**CD4 cell count** (aCD4<200)						
CD4: [200–350]		−0.12				
		(−0.29; 0.05)				
CD4: [350–500]	−0.13	−0.25	−0.13			0.64
	(−0.28; 0.02)	(−0.43; −0.06)*	(−0.29; 0.04)			(0.39; 1.04)
**Marital status (** aSingle)						
Live with spouse/partner	0.12		0.18			
	(−0.02; 0.26)		(0.03; 0.34)*			
Widow(er), Divorced, Separated		−0.16				
		(−0.35; 0.02)				
**Female vs. Male**	0.13	0.17	0.15	0.14	1.31	
	(−0.00; 0.27)	(0.01; 0.33)*	(−0.01; 0.30)	(0.02; 0.26)*	(0.95; 1.81)	
**Income** (aPoorest)						
Rich					1.35	1.51
					(0.93; 1.97)	(0.95; 2.40)
Richest		−0.19				
		(−0.37; −0.01)*				
**Years taking ART**		0.03				
		(−0.01; 0.07)				
**Taking MMT**			−0.17			
			(−0.42; 0.09)			
**History of drug use**			−0.15			1.45
			(−0.31; −0.01)*			(0.97; 2.16)
**HIV/AIDS Stage** (aAsymptomatic)						
Symptomatic			−0.18			
			(−0.41; 0.04)			
AIDS			−0.17			0.72
			(−0.40; 0.07)			(0.46; 1.13)
**Employment:** Other vs. Unemployed				−0.18	0.59	
				(−0.38; 0.03)	(0.33; 1.04)	
**Religion:** Others vs. None					0.55	
					(0.23; 1.29)	
Constant	9.16	9.22	9.24	9.42		
	(8.77; 9.55)**	(8.77; 9.67)**	(8.76; 9.71)**	(9.07; 9.76)**		

95% confidence intervals in parentheses;

* significant at 5%;

** significant at 1%;

a Reference group.

## Discussion

We developed the SATIS - a specific measure to assess patients' satisfaction with HIV/AIDS care and treatment in the Vietnamese context. Using this instrument, we found a high level of patient satisfaction over three SATIS dimensions, including “Quality and Convenience”, “Availability and Responsiveness”, and “Competence of health care workers”. The measure profile showed that the proportion of complete satisfaction was the highest in health care worker's competence and the lowest in the convenience of services. We determined various factors that influenced patient satisfaction with HIV/AIDS care and treatment services, including levels of the health service administration, socioeconomic status (gender, marital status, and income), duration of ART, CD4 cell counts and drug use behavior.

Our findings are consistent with previous studies, which assessed patient satisfaction with HIV/AIDS care and treatment services. The convenience in service utilization, including waiting time and administrative procedures, was observed to be lower than other service quality dimensions in both developed and developing countries [17], [18], [20], [30], [31]. However, the average scores across three measure domains were larger in our study. Besides, other socio-demographic factors associated with patient satisfaction were similar to findings in other settings. In the rural of China, age and income were also found to be significantly related to patient satisfaction [32]. In France, older patients reported higher complete satisfaction with both health workers and services [25]. Noticeably, our findings have enriched the literature by determining the differences in patient satisfaction with HIV/AIDS treatment across levels of health service administration. HIV/AIDS patients at the provincial and district levels were less satisfied with all three quality dimensions than those taking ART at the central level. This reflects the fact that in resource-scare settings, the shortage in health workforce and poorer facilities at the grassroots level could affect the performance of HIV/AIDS treatment services [33]. Moreover, as a country with large injection-driven HIV epidemics, we identified the lack of services' availability and responsiveness for drug users with HIV/AIDS.

Up to now, this is the first study assessing patient satisfaction with HIV/AIDS care and treatment services in various patient groups across levels of health service administration in Vietnam. The study results provide helpful evidence for further expansion of ART services as well as the realization and implementation of the newly introduced Treatment 2.0 initiative in the country. Firstly, there should be cautious steps in decentralizing and integrating ART services to other services at the grassroots level. Services and patients' flow should be managed in a convenient manner, in order to reduce waiting time, simplify administrative procedures, and improve service linkages, referrals, and collaborations. Secondly, additional attention should be paid for ART patients who were drug users. Drug users may have severe co-morbidities that requires various types of health care services and more frequent use [34]. They are also subject to suboptimal adherence to ART and drug or alcohol-related problems [5], [21], [34], [35]. The decrement in satisfaction of drug users in this study indicates that there was insufficient and less responsive health information for drug users. Decentralizing HIV/AIDS care and treatment services in large injection-driven epidemics, therefore, should include capacity building to health workers and integrative and comprehensive health services. It is important to note that this study was conducted in the period of introducing MMT as a substitution therapy for opioid addiction in Vietnam [36]. MMT was found to be a cost-effective service that could improve HIV/AIDS care and treatment outcomes among drug users [36]. However, the current number of MMT sites is still limited and there were only 11.9% of our sample taking MMT. Integrating MMT with ART in this setting could facilitate treatment adherence and improve treatment outcomes among drug users with HIV/AIDS [35]. One priority area of the *Treatment 2.0* initiative is to engage community in delivering ART service as well as treatment support interventions. Findings of this study highlight the importance of family members in supporting patients during the course of ART. Those patients who are living with their couples or partners are more satisfied with services' availability and responsiveness; probably, due to better communication and easier utilization of HIV/AIDS services, as well as perceived emotional and psychological supports among drug users [34], [37].

The strengths of this study include the use of a standard procedure to develop a contextualized and HIV-specific measure of patient satisfaction with HIV/AIDS care and treatment services. Since, there is no gold standard method of measuring patient satisfaction that might be generalizable across settings; this approach could improve the validity and reliability of our measurement [11]. Moreover, the sample included a sufficient number of patients in three epicenters and geographical areas of Vietnam. Besides, there are some limitations that should be acknowledged. First, the cross-sectional design employed in this study might be less capable for causal inference. In addition, patients at clinics were selected conveniently making the sample not representative for the population of HIV/AIDS patients and limiting the generalizability of the study findings.

In conclusion, the patient satisfaction at HIV clinics in Vietnam was generally high. However, performance of HIV/AIDS services at the grassroots level of the health system should be improved. Potential strategies include capacity building for health workers, integrative and comprehensive service delivery, and engagements of family members in treatment supports. Regular assessment of services quality and patient-reported outcomes and satisfaction should be carried out for ART monitoring and evaluation.

## Supporting Information

Table S1 **Satisfaction with HIV/AIDS Treatment Interview Scale (SATIS).**
(DOCX)
