# Supplementary material for: Patient Satisfaction with HIV/AIDS Care and Treatment in the Decentralization of Services Delivery in Vietnam
Source: PLoS One. 2012 Oct 5;7(10):e46680. doi: 10.1371/journal.pone.0046680 (PMC3465274; doi:10.1371/journal.pone.0046680)
Supplement: Table S1 — Satisfaction with HIV/AIDS Treatment Interview Scale (SATIS). (DOCX) [file pone.0046680.s001.docx]

**Table S1: SATISFACTION WITH HIV/AIDS TREATMENT INTERVIEW SCALE (SATIS)**

The following questions ask how you satisfied with the quality of HIV/AIDS care and treatment services you have received in this clinic. There are 10 criteria in a band score of (0->10), where “0” indicates the worst quality and condition and “10” indicates the best quality and condition. I will read out each criterion for you to rate. If you are unsure about which response to give to a question, the first response you think of is often the most appropriate one.

|  | Question | Score |
| --- | --- | --- |
| 1 | How would you rate the overall quality of HIV/AIDS treatment services delivery you have received? |  |
| 2 | How satisfied are you with your access to information and guidance on hospital services and procedures? |  |
| 3 | How satisfied are you with the consultation, explanation, and guidance you have received from doctors, nurses and other health care workers? |  |
| 4 | How would you rate the convenience in check-up booking, waiting time, and administrative procedure you have experienced? |  |
| 5 | How would you rate the convenience in using medical services, such as laboratory tests, referrals, and specialized services? |  |
| 6 | How would you rate the inter-professional and inter-departmental collaborations in this clinic? |  |
| 7 | How would you rate the competency of health care workers? |  |
| 8 | How satisfied are you with the responsiveness of health care workers to your questions and requests? |  |
| 9 | How satisfied are you with the availability of your needed health care services? |  |
| 10 | How satisfied are you with the medical confidentiality and respect of patients’ privacy in this clinic? |  |

Version 1. October, 2011.

Hanoi, Vietnam.
